# Neuroprotective Effects of Curcumin on IL-1β-Induced Neuronal Apoptosis and Depression-Like Behaviors Caused by Chronic Stress in Rats

**DOI:** 10.3389/fncel.2018.00516

**Published:** 2019-01-07

**Authors:** Cuiqin Fan, Qiqi Song, Peng Wang, Ye Li, Mu Yang, Shu Yan Yu

**Affiliations:** ^1^Department of Physiology, School of Basic Medical Sciences, Shandong University, Jinan, China; ^2^Shandong Provincial Key Laboratory of Mental Disorders, School of Basic Medical Sciences, Shandong University, Jinan, China

**Keywords:** interleukin-1β, apoptosis, inflammation, curcumin, depression

## Abstract

Depression is suggested to be a neuropsychiatric disease resulting from neuroinflammation within specific brain regions. Curcumin, a potential neuroprotective agent extracted from curcuma loga, exerts antidepressant-like effects in various animal models of depression. However, the underlying mechanisms, in particular whether curcumin may exert neuroprotection through suppression of inflammatory pathway activity in depression remains largely unknown. In the present study, we examined the molecular events of curcumin as related to its capacity for neuroprotection against inflammation-induced neuronal apoptosis and depression-like behaviors in a rat model of depression. Our results show that chronic administration of curcumin (40 mg/kg, i.p., 5 weeks) prior to stress exposure significantly alleviated depression-like behaviors, expression of the proinflammatory cytokine interleukin-1β (IL-1β) and inhibited neuronal apoptosis within neurons of the ventromedial prefrontal cortex (vmPFC). Within the vmPFC of stressed rats, an intracerebral infusion of an RNAi form of IL-1β in adenovirus associated virus (AAV-IL-1β RNAi) significantly ameliorated depression-like behaviors, neuronal apoptosis and reduced phosphorylated-p38 mitogen-activated protein kinase (p-p38 MAPK) expression levels. More important, within the vmPFC of wild type rats, overexpression of IL-1β via intracerebral infusion of AAV-IL-1β induced p38 MAPK phosphorylation and neuronal apoptosis, which could be significantly prevented by chronic treatment of curcumin. Collectively, these findings reveal that curcumin protects against IL-1β-induced neuronal apoptosis, which may be related to the display of depression-like behaviors in stressed rats. Moreover, they provide new insights into the mechanisms and therapeutic potential for curcumin in the treatment of inflammation-related neuronal deterioration in this disorder.

## Introduction

Depression is one of the most prevalent psychiatric disorders with complex pathogenesis. Previous research has implicated several mechanisms including altered monoaminergic and glutamatergic systems, increased inflammation, HPA axis abnormalities, and decreased neurogenesis and neuroplasticity (Dean and Keshavan, 2017). However, these findings are not present in every patient, which revealed the diversification of pathophysiology in depression that urgently needed to elucidate. Increasing evidence has accrued which indicates that neuroinflammation plays a critical role in the pathogenesis of various neurological disorders including neurodegenerative diseases (Tan et al., 2013; Tuon et al., 2015), stroke (Zhang N. et al., 2014; Kim et al., 2015) and depression (Dowlati et al., 2010; Alcocer-Gomez et al., 2015). However, the pathophysiological mechanisms involved with these disorders are not fully understood. Recently, results from a number of studies have demonstrated that activation of pro-inflammatory factors, such as interleukin-1β (IL-1β), interleukin-6 (IL-6), and tumor necrosis factor-a (TNF-α) may act as a significant factors in the neuronal damage associated with major depressive disorder (MDD) (Maes et al., 2012; Rawdin et al., 2013). In contrast, antidepressants, such as the classic selective serotonin reuptake inhibitor (SSRI), fluoxetine, exert an anti-inflammatory effect via down-regulating microglial activation (Lee et al., 2015; Du et al., 2016). These results provide new insights into potential avenues of investigation regarding antidepressant therapies through their capacity to provide neuroprotection against the inflammation-induced neuronal deterioration that is associated with depression.

Herbal medicines, with their neuroprotective effects and low levels of side effects, have become attractive pharmacological tools in the treatment of various neurological disorders (Wąsik and Antkiewicz-Michaluk, 2017; Maiti and Dunbar, 2018). Curcumin, a natural polyphenolic compound extract from curcuma longa, has been shown to exert a variety of beneficial effects, including anti-inflammatory, anti-tumor, immunomodulatory and neuroprotective activities (Venigalla et al., 2015; Daverey and Agrawal, 2016, 2018). Recently, the potential antidepressant effects of curcumin have been recognized owing to its effectiveness in preventing the genesis of depression-like behavioral phenotypes in various animal models of depression (Andrade, 2014; Lopresti et al., 2014; Zhang L. et al., 2014). Curcumin was reported to reduce levels of inflammatory markers such as TNF-α, NF-κB, IL-1β, iNOS and COX-2 as induced by doxorubicin in rats (Benzer et al., 2018). However, detailed characterizations of the neuronal mechanisms underlying the antidepressant-like effects of curcumin, in particular whether it exhibits neuroprotective effects via inhibiting the activity of inflammatory processes which can lead to neuronal damage in depression remains largely unknown.

Inflammatory cytokines, especially IL-1β, are considered as important pro-apoptotic factors involved with the progression of neurological disorders (Tsai, 2017). IL-1β may contribute to neurofibrillary pathology in Alzheimer’s disease (AD) through activation of the p38 MAPK pathway in cortical neurons (Li et al., 2003), and may also contribute to cortical axon developmental disorders and synaptic deficits through activation of p38 MAPK signaling pathway in septic neonatal rats (Han et al., 2017). The p38 MAPK signaling pathway transduces signals from the cell membrane to the nucleus and, in this way, participates in cell cycle, apoptosis and proliferation (Eriksson et al., 2017). For example, p38 MAPK is crucial for Caspase 3 activation and thus induces neuronal cell apoptosis in the cerebral ischemia-reperfusion injury model (Li and Ai, 2017). However, whether IL-1β up-regulates p38 phosphorylation and thus triggers neuronal apoptosis to promote depression-like behaviors in the chronic unpredictable mild stress (CUMS)-induced animal model requires further investigation.

It should be noted out that not all depressed patients have increased inflammation, previous study reported that the increased inflammation is mainly present in a subgroup of depressed patients who exposure to stress early in childhood or even *in utero*, and these depressed patients is less likely to respond to conventional antidepressants (Pariante, 2017). This shows that many important future questions still exist. What are the epigenetic mechanisms by which chronic stress induce a long-term trajectory of neuroinflammation? How do anti-inflammatories relieve some depressive phenotypes, and whether the anti-inflammatory effects of many herbal medicines serve as the underlying mechanisms of their antidepressant effects?

Therefore, in the present study, we investigated the involvement of inflammation-induced neuronal apoptosis in depression, and whether treatment with curcumin could prevent the neuronal apoptosis and depression-like behaviors induced by inflammation in CUMS-exposed rats. To further explore the underlying mechanisms of the neuroprotective, and thus antidepressant-like effects of curcumin, the regulatory roles of the pro-inflammatory cytokine, IL-1β, upon neuronal apoptosis and expression of apoptosis-related proteins that accompany depression were examined within the vmPFC, a site that represents a critical brain region involved in the pathogenesis of depression in the animal model.

## Materials and Methods

### Animals

Male Wistar rats weighing 240–260 g were obtained from the Experimental Animal Centre of Shandong University. All experiments were approved by the Shandong University Animal Care and Use Committee and were conducted in accordance with the National Institutes of Health Guide for the Care and Use of Laboratory Animals. Rats were allowed to acclimatize to the laboratory environment for 7–8 days prior to use in the experiments. All efforts were made to minimize the pain and numbers of the animals used in these experiments.

### Drugs and Treatment

Curcumin (Sigma, St. Louis, MO, United States) was prepared with use of 0.1% dimethyl sulfoxide (DMSO, Sigma, St. Louis, MO, United States) at a concentration of 10 mg/ml. Due to the poor bioavailability of curcumin when applied oral, we chose a 40 mg/kg intraperitoneal injection regimen as based upon previous results (Bhutani et al., 2009). Curcumin or DMSO was administered daily via an intraperitoneal (i.p.) injection at 30 min prior to a stress exposure that continued for 5 weeks. For CUMS depression model assessment, rats were randomly allocated to one of the following four groups (*N* = 18/group): (a) control (non-stressed group), (b) CUMS, (c) curcumin treatment (40 mg/kg) followed by CUMS, (d) vehicle treatment (0.1% DMSO, 5 ml/kg) followed by CUMS.

### CUMS Procedure

**FIGURE 1:**
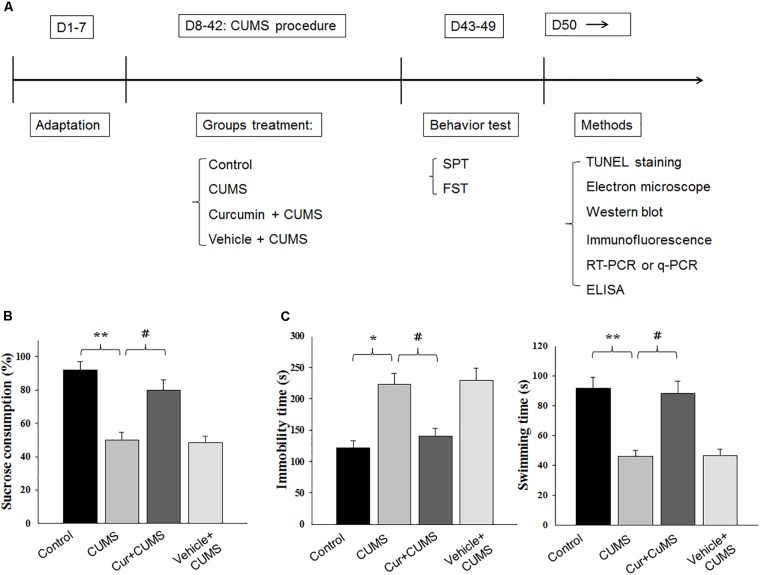
Curcumin rescues CUMS-induced depression-like behaviors in rats. **(A)** Experimental design: schematic figure of the treatment protocol. **(B)** Chronic treatment of curcumin (40 mg/kg) reversed the decreases in percent of sucrose consumption of CUMS-exposed rats. **(C)** Curcumin treatment decreased immobility times and increased swimming times of CUMS-exposed rats in forced swim test. All data are presented as means ± SEM (*N* = 18/group). ^∗^*P* < 0.05, ^∗∗^*P* < 0.01, ^∗∗∗^*P* < 0.001 CUMS vs. Control group (non-stressed). #*P* < 0.05, ##*P* < 0.01, ###*P* < 0.001 Cur + CUMS vs. CUMS group (Cur, Curcumin).

The CUMS procedure was performed according to procedures described previously with minor modifications (Mao et al., 2009). Rats in the non-stressed control group were housed in groups of four per cage in the colony room while rats in the stressed-groups were housed individually in a separate colony room and subjected to a daily stress regime over the 5-week period. Unpredictable mild stressors were applied in a variable sequence that included overnight illumination, 24 h food deprivation followed by 24 h water deprivation, 5 min cold swimming (4°C), cage shaking (2 h), physical restraint (2 h), wet bedding (24 h) and 1 min tail pinch (1 cm from the end of the tail). Each of these stressor episodes were applied daily to each rat in a random order (Figure 1A).

### Stereotaxic Injection of the AAV Virus

For AAV viruses, the HBAAV2/9-r-IL-1β-GFP virus (AAV-IL-1β, Hanbio Biotechnology, Shanghai, China) was used to overexpress IL-1β in the vmPFC and the HBAAV2/9-r-IL-1β shRNAi-GFP virus (AAV-IL-1βi, Hanbio Biotechnology, Shanghai, China) to block IL-1β in the vmPFC. In this series of experiments, rats were randomly allocated to one of the following groups (*N* = 18/group): (a) wild type (non-stressed and non-injected group), (b) wild type + AAV-control (GFP-Cre construct), (c) wild type + AAV-IL-1β, (d) stressed, (e) stressed + AAV-control (GFP-Cre construct), (f) stressed + AAV-IL-1β RNAi and (g) stressed + AAV- IL-1β RNAi + curcumin. For viral injections, rats were deeply anesthetized with sodium pentobarbital (150 mg/kg, i.p.) and placed in a stereotaxic frame (Stoelting, United States). The vmPFC injection site was determined according to coordinates of the Rat Brain Atlas (from bregma: AP, +3.24 mm; ML, ±0.5 mm; DV, -4.8 mm). Rats were infused bilaterally with 1–1.5 μl of purified and concentrated AAV virus ( ∼10^12^ infection units per ml) using microinjection pump (Stoelting, United States) at a rate of 150 nl/min. The microelectrode remained in the injection site for at least 5 min after infusion and was then slowly withdrawn. Behavioral experiments or biochemical assays were performed at a minimum of 14 days after viral infusion into the vmPFC. The injection sites were examined after the behavioral tests and only data from rats with correct injection site placements within the vmPFC were included in the analyses.

### Behavioral Tests

Behavioral tests were conducted after 5 weeks of CUMS treatment in the following sequence.

#### Sucrose Preference Test

The sucrose preference test was performed to assess anhedonia in rats as described previously with minor modifications (Mao et al., 2009). In the initial adaption phase, rats were placed individually in cages with two bottles of sucrose solution (1%, w/v) for a 24 h period; one bottle was then replaced with tap water for the second 24 h period. In the test phase, rats were deprived of water and food for 24 h and then permitted 3 h of free access to the two bottles, one filled with 100 ml of 1% sucrose solution and the other 100 ml of tap water. The consumption of the sucrose solution and tap water were measured and the sucrose preference was defined as sucrose consumption/[water consumption + sucrose consumption] × 100% during the 3 h test.

#### Forced Swim Test

On the day after the sucrose preference test, the forced swim test was conducted to assess “behavioral despair” in rats as described previously (Porsolt et al., 1977; Duman et al., 2007). In the training session, rats were placed individually in a glass cylinder (Temperature: 25°C, height: 80 cm, diameter: 30 cm) filled with 40 cm of water for 15 min of forced swimming. The test session was performed 24 h later. Rats were individually placed in the cylinder for a 5 min period, during which immobility and swimming times were recorded. The immobility time was defined as floating with only small movements necessary to maintain their head above the water.

### Electron Microscopy Analysis

Two days after behavioral testing, brain samples from four rats per group were prepared for transmission electron microscopy (TEM). The vmPFC (1 mm × 1 mm × 1 mm ) was carefully dissected and primary-fixed in buffered glutaraldehyde 2.5% for a minimum of 4 h at 4°C. The tissue was then post-fixed in 1% buffered osmium tetroxide for 2 h. After an ascending series of graded ethanol dehydrations, the tissue was infiltrated with a mixture of one-half propylene oxide overnight and then embedded in resin. Ultrathin sections (70 nm) were cut and stained with 4% uranyl acetate for 20 min followed by 0.5% lead citrate on copper grids for 5 min. Ultrastructure of the vmPFC was then observed with use of TEM (Philips Tecnai 20 U-Twin, Holland). A minimum of 30 micrographs per rat were randomly selected for analysis.

### TUNEL Staining

Two days after behavioral testing, paraffin embedded sections of the vmPFC were sampled to measure TUNEL positive cells with use of the In Situ Cell Death Detection Kit (Roche, Germany) according to the manufacturer’s instructions. Briefly, 5 μm sections were dewaxed and rehydrated according to standard protocols consisting of incubating with protease K (10 μg/ml) at room temperature for 15 min. Sections were then incubated with the TUNEL reaction mixture at 37°C for 1 h followed by incubation with 50 μl DAB Substrate (DAKO, Denmark) for 10 min at room temperature. Images were captured using light microscopy (Nikon, Japan). The number of TUNEL positive neurons was counted within three different fields (500 × 500 μm grids) per section and calculations were performed within 12 randomly selected fields per rat. Data were expressed as the ratio of positive cells obtained relative to that of the control group.

### Immunofluorescence Assay

Rats were anesthetized with sodium pentobarbital (150 mg/kg, i.p.) and perfused with 4% paraformaldehyde (PFA). Brains were collected and post-fixed overnight at 4°C followed by a graded dehydration. Brain samples were then cut into serial coronal frozen sections (30 μm). Sections were incubated with the primary polyclonal rabbit anti-ionized calcium binding adaptor molecule-1 (Iba-1) (1:500, WAKO, Japan) and rabbit anti-glial fibrillary acidic protein (GFAP) (1:100, Proteintech, United States) followed by the fluorescent-conjugated secondary antibody (goat anti-rabbit IgG, 1:200; Sigma-Aldrich). Images were captured with use of a scanning laser confocal microscope (LSM780, Carl Zeiss, Germany). At least six to eight representative images were taken from each rat for analysis by Image-Pro plus 6.0 software. The number of Iba-1/DAPI double positive cells and GFAP/DAPI double positive cells were determined in each section (*N* = 6 rats/group). The values were expressed as the ratio of double positive cells relative to that of the control group.

### Reverse Transcription PCR

Total RNA was isolated and extracted from samples of the vmPFC using a TRIpure Reagent Kit (Invitrogen, United States) and reverse-transcribed into cDNA with use of the HIScript II Q RT SuperMix Kit for qPCR (+gDNA wiper) (TaKaRa, Japan). Reverse transcription was then conducted using a 2xEasy Taq PCR SuperMix Kit for PAGE (TransGen, China). PCR products were assessed by electrophoresis on a 3% agarose gel and were analyzed using the Gel Image Analysis System (Bio-Rad, United States). Levels of targeted mRNA were normalized to the housekeeping gene GAPDH. Real-time PCR was performed with use of the Bio-Rad IQ5 Real Time PCR System (Bio-Rad, United States). A relative fold change in expression of the gene was determined using the RQ = 2^-Ct^ method by Bio-Rad IQ5 Software (Bio-Rad, United States). The primers used for PCR amplification were as follows: (F, forward; R, reverse)

IL-1β (F): GGGATGATGACGACCTGC;
(R): CCACTTGTTGGCTTATGTT;
Bcl-2 (F): GGATCCAGGATAACGGAGGC;
(R): ATGCACCCAGAGTGATGCAG;
Bak (F): TCTTCAAACTGCTGGGCCATT;
(R): CTTGTCACCTGCCTGACTGCT;
Caspase3 (F): GGAGCTTGGAACGCGAAGAA;
(R): ACACAAGCCCATTTCAGGGT
Caspase9 (F): CAAGAAGAGCGGTTCCTGGT;
(R): CAGAAACAGCATTGGCGACC;
GAPDH (F): AGTGCCAGCCTCGTCTCATA;
(R): GGTAACCAGGCGTCCGATAC.

### Enzyme Linked Immunosorbent Assay (ELISA)

Concentrations of IL-1β, Bcl-2, Bcl-2, Caspase 3 and Caspase 9 were measured with use of cytokine ELISA assays according to the manufacturer’s instructions (Abcam Co., United Kingdom). Total protein isolated from vmPFC tissue samples was determined with use of the BCA assay (Thermo Fisher, Waltham, MA, United States). For serum IL-1β detection, all blood samples were obtained via venous collection. Equal amounts of diluted samples were added to each well of the ELISA kits (96 well) coated with the appropriate antibody. Data were displayed as cytokine (pg) vs. total protein (mg) (mean ± SEM). *N* = 6–8 animals per group.

### Western Blot Analysis

Two days after behavioral testing, rats were anesthetized with sodium pentobarbital (150 mg/kg, i.p.), and the vmPFC was carefully dissected. Thirty μg of proteins per lane were electrophoretically separated on SDS-PAGE gels and transferred onto PVDF membranes which were then incubated overnight at 4°C with the appropriate antibodies. Primary antibodies used included polyclonal rabbit anti-p38 MAPK (1:1000, CST-9212S, Cell Signaling Technology, Beverly, MA, United States), anti-phospho-p38 MAPK (1:500, CST-9211S, Cell Signaling Technology, Beverly, MA, United States) and anti-β-actin (1:8000) (SC-47778, Santa Cruz Biotechnology, Santa Cruz, CA, United States). The secondary antibody was horseradish peroxidase-conjugated antibody (1:5000, SC-2030, Santa Cruz Biotechnology, Santa Cruz, CA, United States). The blots were detected using an enhanced chemiluminescence detection kit (GE Healthcare, Buckinghamshire, United Kingdom) and protein band densities were quantified using Image-J software (NIH, Scion Corporation, Frederick, MD, United States). Final data were expressed as a percent difference from that of the control group.

### Data Analysis

All statistical procedures were performed on SPSS version 13.0. One-way or two-way analysis of variance (ANOVA) was used to establish differences among groups followed by the Tukey’s test for post-hoc comparisons. The data were expressed as mean ± SEM of at least four individual animals. *P* < 0.05 was required for differences to be considered statistically significant.

## Results

### Curcumin Treatment Rescued Depression-Like Behaviors Induced by CUMS Exposure

The effect of curcumin treatment on sucrose preference in CUMS-exposed rats is presented in Figure 1. One-way ANOVA revealed that there was an overall statistically significant difference in the percent of sucrose consumption among the four groups [*F*_(3,68)_ = 19.23, *P* < 0.05] (Figure 1B). *Post hoc* analysis indicated that a 5-week period of CUMS exposure significantly reduced the percent of sucrose consumption in rats as compared with that of the non-stressed control group (*P* < 0.01). Chronic pretreatment with curcumin (40 mg/kg daily) significantly increased the percent of sucrose consumption in CUMS-exposed rats (*P* < 0.05). No statistically significant differences were obtained between the vehicle-treated CUMS-exposed and CUMS-exposed control groups with regard to percent of sucrose consumption (*P >* 0.05).

Another method to assess antidepressant-like effects is the forced swim test. As shown in Figure 1C, results of the one-way ANOVA revealed an overall significant difference among the four groups with regard to both immobility times [*F*_(3,68)_ = 18.12, *P* < 0.05] and swimming times [*F*_(3,68)_ = 15.87, *P* < 0.05]. *Post hoc* analysis indicated that 5-weeks of CUMS exposure significantly increased immobility times (*P* < 0.05) and decreased swimming times in rats as compared to the non-stressed control group (*P* < 0.01). In contrast, rats pretreated with curcumin showed significantly decreased immobility times and significantly increased swim times as compared to CUMS-exposed control group (*P* < 0.05, for both). Overall, the main findings of both behavioral tests indicating that chronic curcumin treatment of CUMS rats exerted antidepressant-like effects in this animal model of depression.

### Curcumin Treatment Reduced CUMS-Induced Glial Activation and IL-1β Overexpression Within the vmPFC

**FIGURE 2:**
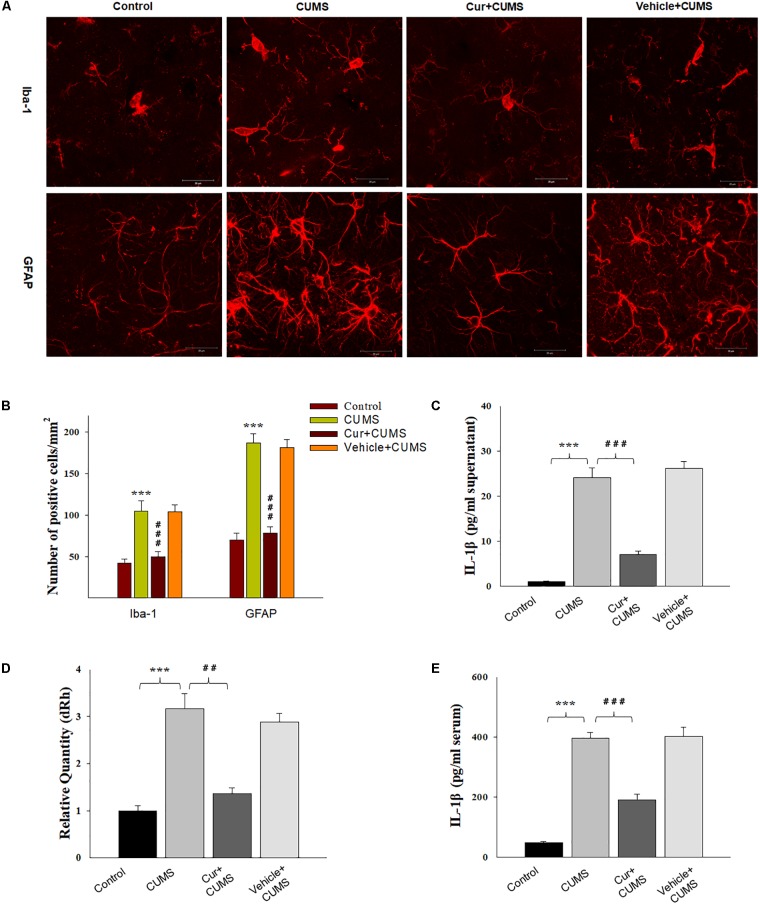
Curcumin attenuated glial activation and IL-1β expression induced by CUMS-exposure. **(A)** CUMS exposure significantly increased the number of Iba1 positive microglial cells (top) and GFAP positive cells (bottom) within the vmPFC. **(B)** Histograms showing the glia activation were significantly attenuated by curcumin pretreatment. **(C)** ELISA assays showed that CUMS exposure increased IL-1β protein expression within the vmPFC, effects which were ameliorated by curcumin. **(D)** Real-time quantitative PCR assays showed that CUMS exposure increased IL-1β mRNA expression within the vmPFC, effects which were ameliorated by curcumin. **(E)** ELISA assays showed that CUMS exposure increased serum IL-1β expression, effects which were ameliorated by curcumin. Data were presented as the means ± SEM (*N* = 6/group). ^∗^*P* < 0.05, ^∗∗^*P* < 0.01, ^∗∗∗^*P* < 0.001 CUMS vs. Control group; #*P* < 0.05, ##*P* < 0.01, ###*P* < 0.001 Cur + CUMS vs. CUMS group (Cur, Curcumin).

The results of immunofluorescence assay showed that was significantly different among the four groups with regard to the number of Iba-1^+^ microglia [*F*_(3,20)_ = 17.63, *P* < 0.001] and GFAP positive astroglia [*F*_(3,20)_ = 18.05, *P* < 0.001] within the vmPFC region (Figures 2A,B). *Post hoc* analysis indicated that the 5-weeks of CUMS exposure significantly increased the number of activated vmPFC microglia and astroglia as compared to the non-stressed rats (*P* < 0.001, for both). These changes in glia number were significantly reversed by chronic pretreatment with curcumin (*P* < 0.001, for both). As IL-1β is a critical pro-inflammatory factor in brain, we next examined whether curcumin could modulate IL-1β expression within the vmPFC of CUMS-exposed rats. Significant differences were obtained among the four groups with regard to the expression of IL-1β [qPCR: *F*_(3,20)_ = 17.16; *P* < 0.001; ELISA: *F*_(3,20)_ = 16.85; *P* < 0.001] (Figures 2C,D). *Post hoc* analysis showed that after 5-weeks exposure to CUMS, the mRNA and protein expression levels of IL-1β within the vmPFC were significantly increased (*P* < 0.001). Moreover, the level of serum IL-1β were significantly different between these groups [*F*(3,20) = 17.16; *P* < 0.001] (Figure 2E). *Post hoc* analysis showed that the serum IL-1β level in this depression animal model was significantly increased (*P* < 0.001). These increases were significantly reversed by pre-administration of curcumin.

### Curcumin Treatment Attenuated CUMS-Induced Apoptosis in the vmPFC

**FIGURE 3:**
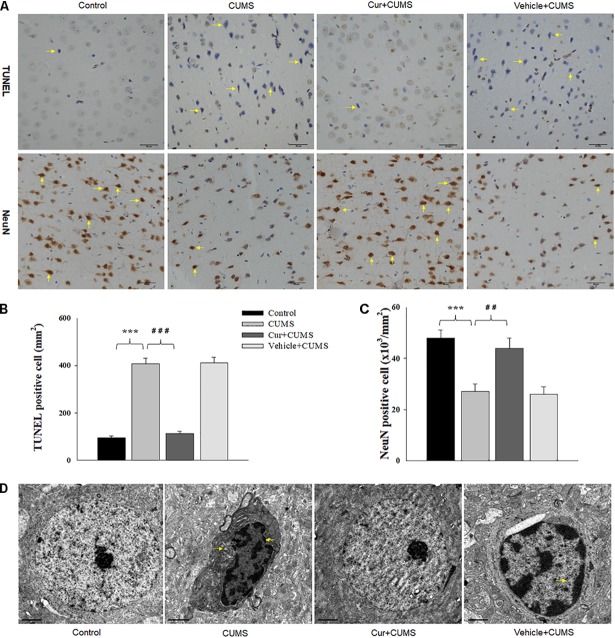
Curcumin ameliorated neuronal apoptosis within the vmPFC as induced by CUMS exposure. **(A)** Curcumin treatment decreased the number of TUNEL positive apoptotic cells (top, indicated by the arrows) and NeuN positive cells (bottom, indicated by the arrows) in the vmPFC of CUMS-exposed rats. Scale bar is 20 μm. **(B)** Histograms showing the increased TUNEL positive cells were significantly attenuated by curcumin pretreatment. **(C)** Histograms showing the decreased NeuN positive cells was significantly attenuated by curcumin pretreatment. **(D)** Representative electron micrograph of vmPFC neuronal ultrastructure. Arrows indicate nuclear chromatin aggregation, condensation and margination. Scale bar is 2 μm. Curcumin significantly ameliorated apoptosis of vmPFC neurons in CUMS-exposed rats. *N* = 6/group. ^∗^*P* < 0.05, ^∗∗^*P* < 0.01, ^∗∗∗^*P* < 0.001 CUMS vs. Control group (non-stressed). #*P* < 0.05, ##*P* < 0.01, ###*P* < 0.001 Cur + CUMS vs. CUMS group.

As shown in Figure 3A, one-way ANOVA results of the TUNEL Staining assay revealed that there was an overall statistically significant difference in the density of apoptotic cells among the four groups [*F*_(3,20)_ = 16.52, *P* < 0.001]. TUNEL positive labeled cells were found to be particularly prevalent within the vmPFC of CUMS-exposed rats. Post-hoc analysis indicated that chronic CUMS-exposure significantly increased the density of apoptotic cells within vmPFC regions (*P* < 0.001), while chronic curcumin pretreatment markedly inhibited this massive CUMS-induced loss and apoptosis of neurons in the vmPFC area of CUMS rats (Figure 3B). In addition, immunohistochemistry assays showed that NeuN positive labeled cells were also significantly decreased within the vmPFC regions after chronic CUMS-exposure (*P* < 0.001) (Figures 3A,C), while curcumin pretreatment markedly inhibited this massive cell loss in the vmPFC area of CUMS-exposed rats (*P* < 0.01).

As nuclear chromatin condensation and margination represent the main changes observed within apoptotic cells, we next examined the neuronal ultrastructure in the vmPFC using TEM. The vmPFC neurons within the CUMS-exposure group showed strong staining of the condensed portion of their chromosomes accompanied with nuclear chromatin margination, aggregation and condensation, all of which indicate apoptotic morphological changes induced by CUMS exposure. In contrast, these apoptotic morphological changes were obviously alleviated by chronic pretreatment with curcumin (Figure 3D).

### Curcumin Rescued Depression-Like Behaviors Caused by IL-1β Overexpression in vmPFC

**FIGURE 4:**
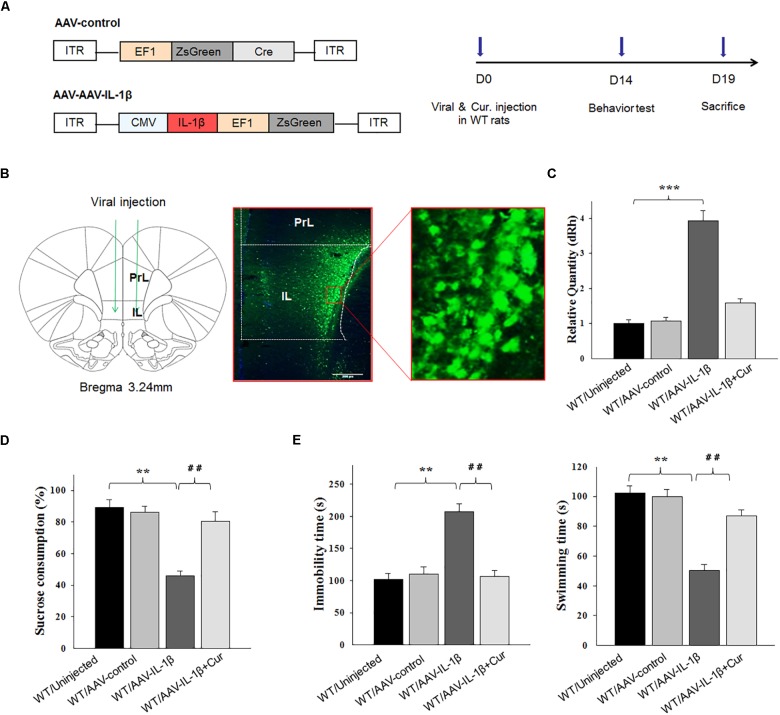
Overexpression of IL-1β within the vmPFC leads to depression-like phenotypes in normal wild type rats. **(A)** Left: Schematics of AAV vectors engineered to overexpress a control construct or IL-1β. ITR, inverted terminal repeats; EF1, ZsGreen promoter; CMV, IL-1β promoter. Right: Experimental paradigm for behavioral biochemical testing of rats infected with the virus. **(B)** Illustration of viral infusion of AAV-IL-1β into the vmPFC. PrL, prelimbic cortex; IL, infralimbic cortex. Scale bar is 100 μm. **(C)** Estimation of βCaMKII overexpression efficiency after viral constructs injection. **(D,E)** Behavioral effects of over expressing viral AAV-IL-1β in the vmPFC. *N* = 12 in all groups. ^∗^*P* < 0.05, ^∗∗^*P* < 0.01, ^∗∗∗^*P* < 0.001 WT + AAV-IL-1β vs. WT rats. #*P* < 0.05, ##*P* < 0.01, ###*P* < 0.001 WT + AAV-IL-1β + Cur vs. WT + AAV-IL-1β (WT, wild type).

As IL-1β is a potential modulator of cell apoptosis, to further explore whether curcumin treatment could alleviate CUMS-induced apoptosis via suppressing IL-1β expression, a AAV-IL-1β virus was constructed (Figure 4A) and infused bilaterally into the vmPFC of non-stressed normal wild type rats to overexpress IL-1β, followed by treatment with curcumin for 14 days (Figure 4B). After estimating the overexpression efficiency (*P* < 0.001, Figure 4C), the behavioral tests were then conducted. Results revealed that significant differences were present regarding the percent of sucrose consumption [*F*_(3,44)_ = 16.19; *P* < 0.01] (Figure 4D), immobility times [*F*_(3,44)_ = 14.98; *P* < 0.01] and swimming times [*F*_(3,44)_ = 15.47, *P* < 0.01] among the four groups (Figure 4E). *Post hoc* analysis showed that an overexpression of IL-1β within the vmPFC significantly decreased sucrose consumption and increased immobility times as compared with rats receiving an AAV-vector control injection (*P* < 0.01). In contrast, 14-days of curcumin treatment following AAV-IL-1β virus injection significantly reversed these depression-like behaviors induced by overexpression of IL-1β within the vmPFC (*P* < 0.01). These results indicate that curcumin may exert antidepressant-like effects via suppression of the IL-1β pathway.

### Curcumin Attenuated Neuronal Apoptosis in vmPFC Caused by IL-1β Overexpression

**FIGURE 5:**
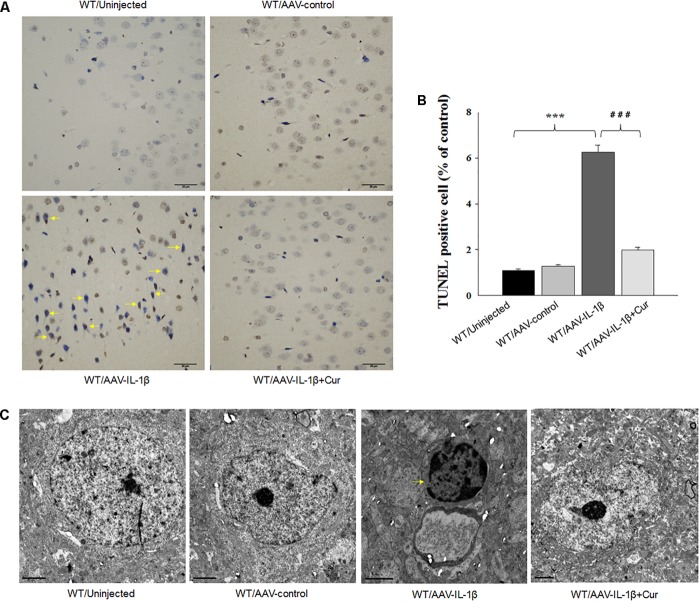
Overexpression of IL-1β within the vmPFC leads to neuronal apoptosis within the vmPFC of normal wild type rats. **(A)** TUNEL positive apoptotic cells (indicated by the arrows) in vmPFC regions infected with AAV-IL-1β. **(B)** Histograms showing the increased TUNEL positive cells caused by AAV-IL-1β infusion were significantly attenuated by curcumin treatment. **(C)** Nuclear chromatin abnormalities in vmPFC neurons infected with AAV-IL-1β. Arrows indicate nuclear chromatin aggregation, condensation and margination. *N* = 6/group. ^∗^*P* < 0.05, ^∗∗^*P* < 0.01, ^∗∗∗^*P* < 0.001 WT + AAV-IL-1β vs. WT rats. #*P* < 0.05, ##*P* < 0.01, ###*P* < 0.001 WT + AAV-IL-1β + Cur vs. WT + AAV-IL-1β.

Therefore, we next investigated whether the overexpression of IL-1β in the vmPFC, which induces neuronal apoptosis, could be prevented by curcumin treatment. One-way ANOVA results from the TUNEL Staining assay showed an overall statistically significant difference in the density of apoptotic cells among these four groups [*F*_(3,20)_ = 15.91, *P* < 0.001] (Figures 5A,B). *Post hoc* analysis revealed that IL-1β overexpression significantly increased the density of apoptotic cells within vmPFC regions (*P* < 0.001). Furthermore, a high-density chromatin margination was present under the nuclear membrane in the group treated with an AAV-IL-1β injection as revealed with TEM (Figure 5C). Moreover, curcumin treatment dramatically attenuated the apoptotic cells (*P* < 0.001) and apoptotic related morphological changes induced by overexpression of IL-1β. These results indicate that the pro-inflammatory cytokine, IL-1β, likely produced the substantial increases in apoptotic cells within the vmPFC in this animal model of depression, effects which could be ameliorated by curcumin treatment.

### Curcumin Attenuated Apoptosis-Related Factors Expression Caused by IL-1β Overexpression

**FIGURE 6:**
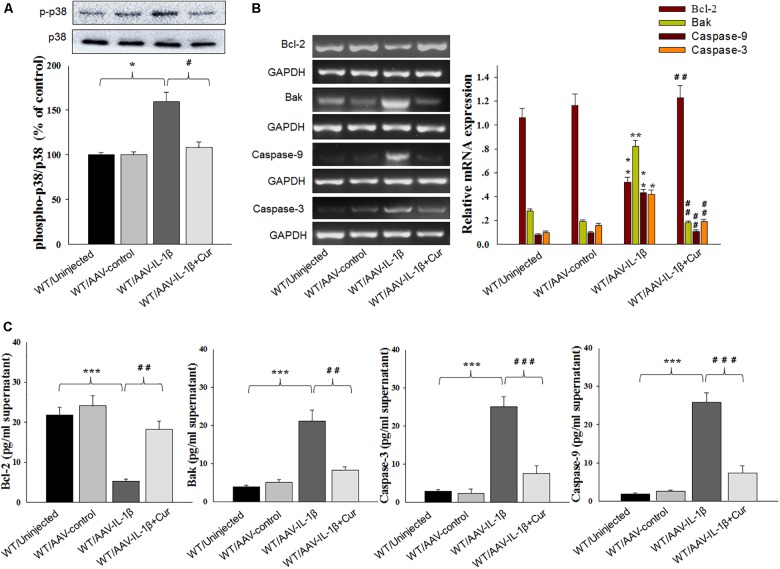
Curcumin attenuated apoptosis-related factors expression caused by IL-1β overexpression. **(A)** Phosphorylation levels of p38 MAPK in the vmPFC infected with the AAV-IL-1β virus. **(B)** RT-PCR assays of expression levels of Bcl-2, Bak, Caspase 3 and Caspase 9 in vmPFC neurons infected with AAV-IL-1β. **(C)** ELISA assays of expression levels of Bcl-2, Bak, Caspase 3 and Caspase 9 in vmPFC neurons infected with AAV-IL-1β. *N* = 6/group. ^∗^*P* < 0.05, ^∗∗^*P* < 0.01, ^∗∗∗^*P* < 0.001 WT + AAV-IL-1β vs. WT rats. #*P* < 0.05, ##*P* < 0.01, ###*P* < 0.001 WT + AAV-IL-1β + Cur vs. WT + AAV-IL-1β.

To further substantiate these findings, we examined the effects of IL-1β overexpression on apoptosis-related protein expression levels. Similar to that obtained with morphological results, there was an overall statistically significant difference among the four groups in biochemical markers associated with the apoptotic markers Bcl-2 [RT-PCR: *F*_(3,20)_ = 14.83; *P* < 0.01; ELISA: *F*_(3,20)_ = 15.76; *P* < 0.001], Bak [RT-PCR: *F*_(3,20)_ = 15.18; *P* < 0.01; ELISA: *F*_(3,20)_ = 12.63; *P* < 0.001], Caspase-3 [RT-PCR: *F*_(3,20)_ = 15.04; *P* < 0.01; ELISA: *F*_(3,20)_ = 13.96; *P* < 0.001] and Caspase-9 [RT-PCR: *F*_(3,20)_ = 15.21; *P* < 0.01; ELISA: *F*_(3,20)_ = 12.75; *P* < 0.001] within vmPFC regions (Figures 6B,C). *Post hoc* analysis showed that an overexpression of IL-1β within the vmPFC of non-stressed rats significantly decreased mRNA and protein expression of Bcl-2 and increased expression levels of pro-apoptotic proteins. Notably, curcumin treatment dramatically attenuated the apoptotic related morphological and biochemical changes induced by overexpression of IL-1β (*P* < 0.01). More importantly, we found that IL-1β overexpression also produced an overall statistically significant increase in the phosphorylation of P38 MAPK [*F*_(3,20)_ = 16.83, *P* < 0.05] (Figure 6A), which proposed that p38 MAPK may act as a critical downstream molecule trigger for apoptotic processes in the vmPFC.

### Knocking-Down of IL-1β in vmPFC Rescued Depression-Like Behaviors in Stressed Rats

**FIGURE 7:**
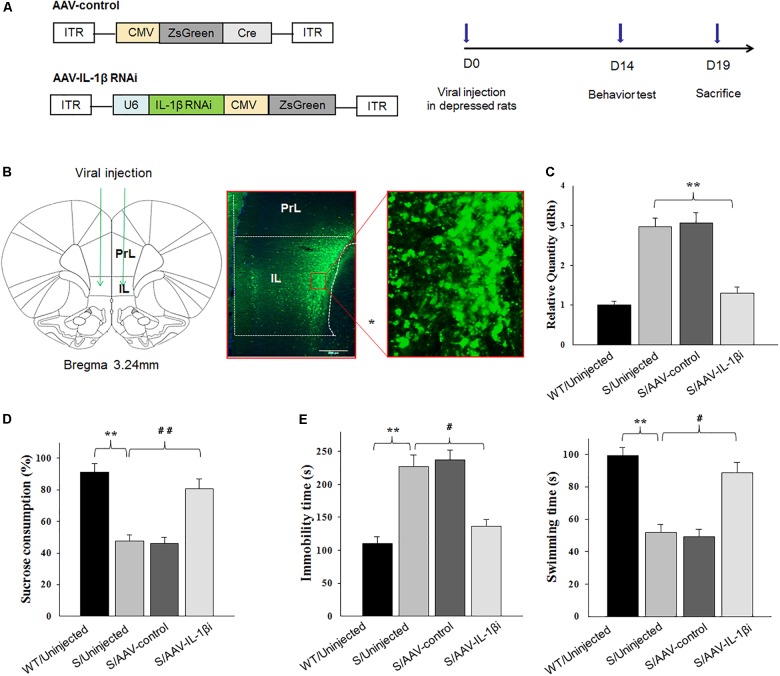
Knocking-down of IL-1β in the vmPFC reduced depression-like phenotypes of stressed rats. In these experiments, prior to virus infusion, rats experienced a 5-week period of CUMS exposure to induce depression-like phenotypes. **(A)** Left: Schematics of AAV vectors engineered to overexpress a control construct or RNAi form of IL-1β. U6, IL-1β shRNA promoter. Right: Experimental paradigm for behavioral testing of rats infected with the virus. **(B)** Illustration of viral infusion of the IL-1β RNAi construct into the vmPFC. Scale bar is 100 μm. **(C)** Estimation of βCaMKII overexpression efficiency after viral constructs injection. **(D,E)** Behavioral effects of expressing AAV-IL-1β RNAi construct within the vmPFC of stressed rats. *N* = 12 in all groups. ^∗^*P* < 0.05, ^∗∗^*P* < 0.01, ^∗∗∗^*P* < 0.001 Stressed rats vs. WT rats. #*P* < 0.05, ##*P* < 0.01, ###*P* < 0.001 S+AAV-IL-1β RNAi vs. Stressed rats (S, stressed).

Next, we used RNA interference (RNAi) to reduce (knock down) IL-1β protein as a means to determine whether a down-regulation or blockade of IL-1β function within the vmPFC would reverse depression phenotypes (Figure 7A). Behavioral testing of depression was then performed at 14-days post-infusion of the small interference RNA sequence form of IL-1β in the AAV virus (AAV-IL-1β-RNAi) in vmPFC of stressed rats (Figures 7B,C). One-way ANOVA revealed an overall statistically significant difference in behaviors related to depression. Specifically, the percent of sucrose consumption [*F*_(3,44)_ = 17.36; *P* < 0.01] (Figure 7D), immobility times [*F*_(3,44)_ = 16.79; *P* < 0.01] and swimming times [*F*_(3,44)_ = 15.16, *P* < 0.01] (Figure 7E) all showed significant differences among the four groups. Post-hoc analysis indicated that infused AAV-IL-1β-RNAi to block IL-1β function in stressed rats produced significant increases in the percent of sucrose consumption in the sucrose preference test and also prevented the “behavioral despair” symptoms (increased immobility times and decreased swimming times in stressed rats) in the forced swim test (*P* < 0.05).

### Knocking-Down of IL-1β in vmPFC Reduced Neuronal Apoptosis in Stressed Rats

**FIGURE 8:**
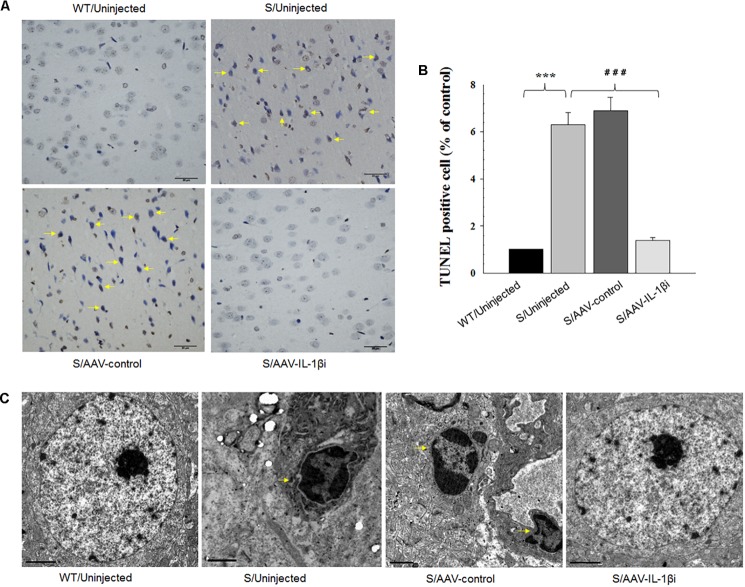
Knocking-down of IL-1β in the vmPFC decreased neuronal apoptosis within the vmPFC of stressed rats. **(A)** TUNEL positive apoptotic cells (indicated by the arrows) in vmPFC regions infected with AAV-IL-1β RNAi construct. **(B)** Histograms showing the decreased TUNEL positive cells caused by AAV-IL-1β RNAi construct infusion. **(C)** nuclear chromatin abnormalities in vmPFC neurons infected with the AAV-IL-1β RNAi construct. Arrows indicate nuclear chromatin aggregation, condensation and margination. *N* = 6/group. ^∗^*P* < 0.05, ^∗∗^*P* < 0.01, ^∗∗∗^*P* < 0.001 Stressed rats vs. WT rats. #*P* < 0.05, ##*P* < 0.01, ###*P* < 0.001 S+AAV-IL-1β RNAi vs. Stressed rats.

**FIGURE 9:**
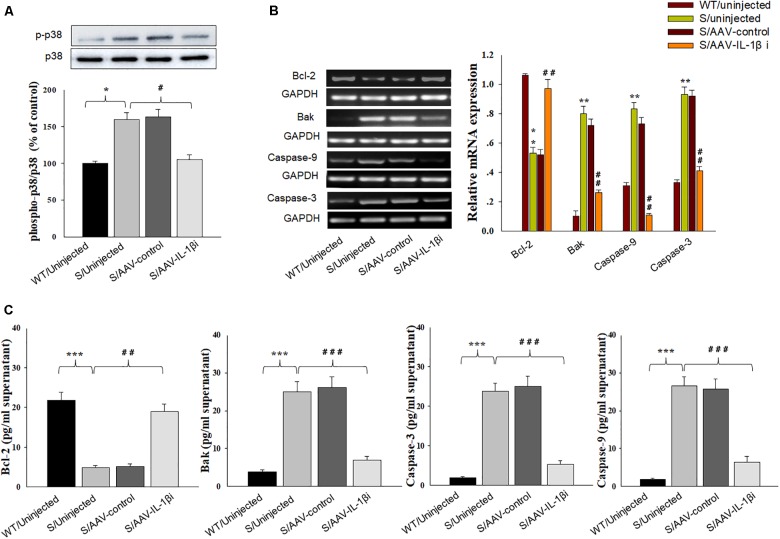
Knocking-down of IL-1β in vmPFC reduced apoptosis-related factors produced by CUMS exposure. **(A)** Phosphorylation levels of p38 MAPK in the vmPFC infected with the AAV-IL-1β RNAi virus. **(B)** RT-PCR assays of expression levels of Bcl-2, Bak, Caspase 3 and Caspase 9 in vmPFC neurons infected with the AAV-IL-1β RNAi construct. **(C)** ELISA assays of expression levels of Bcl-2, Bak, Caspase 3 and Caspase 9 in vmPFC neurons infected with the AAV-IL-1β RNAi construct. *N* = 6/group. ^∗^*P* < 0.05, ^∗∗^*P* < 0.01, ^∗∗∗^*P* < 0.001 Stressed rats vs. WT rats. #*P* < 0.05, ##*P* < 0.01, ###*P* < 0.001 S+AAV-IL-1β RNAi vs. Stressed rats.

In addition to its effects upon depression-like behaviors, we also found that blockage of the IL-1β pathway also produced substantial effects upon amelioration of neuronal apoptosis [*F*_(3,20)_ = 16.39, *P* < 0.001, Figures 8A,B] and nuclear apoptotic morphological changes (Figure 8C) within the vmPFC of stressed rats. To corroborate this result, we examined the modulatory effects of IL-1β on the expression of apoptosis-related proteins. As shown in Figures 9B,C, mRNA and protein expressions of Bcl-2 within the vmPFC differed significantly among the four groups [RT-PCR: *F*_(3,20)_ = 15.07; *P* < 0.01; ELISA: *F*_(3,20)_ = 14.91; *P* < 0.001]. Moreover, one-way ANOVA also revealed that there was an overall statistically significant difference among the four groups with regard to the mRNA and protein expression of pro-apoptosis factors such as Bak [RT-PCR: *F*_(3,20)_ = 14.72; *P* < 0.01; ELISA: *F*_(3,20)_ = 12.96; *P* < 0.001], Caspase-3 [RT-PCR: *F*_(3,20)_ = 12.78; *P* < 0.01; ELISA: *F*_(3,20)_ = 13.26; *P* < 0.001] and Caspase-9 within vmPFC regions [RT-PCR: *F*_(3,20)_ = 15.17; *P* < 0.01; ELISA: *F*_(3,20)_ = 12.39; *P* < 0.001]. *Post hoc* analysis indicated that injection of AAV-IL-1β-RNAi significantly increased the expression of Bcl-2, along with a decrease in expression levels of Bak, Caspase-3 and Caspse-9 within the vmPFC (*P* < 0.01). In addition, one-way ANOVA analysis of P38 MAPK activity within the vmPFC also showed an overall statistically significant difference among the four groups with regard to phosphorylation levels of p38 MAPK [*F*_(3,20)_ = 15.24; *P* < 0.05] (Figure 9A). *Post hoc* analysis indicated that P38 MAPK phosphorylation levels in the vmPFC were significantly reduced in the AAV-IL-1β-RNAi infused stressed groups. When combined with the results obtained from the IL-1β-overexpression experiments, these results provide evidence indicating that suppression of phosphorylated p38 MAPK may contribute to the antidepressant-like behaviors caused by IL-1β knock down.

## Discussion

In the present study, we show that curcumin exerts neuroprotective effects against apoptosis-related depression-like behaviors in a CUMS rat model. Moreover, we found that curcumin ameliorated IL-1β-induced neuronal apoptosis via suppressing the P38 pathway within the vmPFC of CUMS exposed rats. Taken together, these findings suggest that: (1) chronic stress-induced inflammation may contribute to neuronal apoptosis and thus promote depression-like symptoms and (2) the neuroprotectant, curcumin, exhibits antidepressant-like effects via suppressing the pathway involved with inflammation-induced apoptosis within the vmPFC, a brain site associated with depression.

The classical antidepressants currently used for the treatment of depression usually induce many side effects and possess low efficacy (Ereshefsky, 2009). Recent evidence has accrued indicating the potential antidepressant effects of curcumin in various animal models of depression; moreover this agent has attracted considerable attention due to its high safety margins (Zhang et al., 2013; Lopresti, 2017) and its ability to function as a multi-target natural compound which may modulate numerous pathways (Zhou et al., 2011). Within our laboratory we have shown that curcumin prevented neuronal plasticity dysregulation induced by chronic stress via its neuroprotective ability involving MAPK/ERK-dependent brain-derived neurotrophic factor expression (Zhang L. et al., 2014), as well as by modulating the miR-134 signaling pathway (Fan et al., 2018). Furthermore, curcumin has been reported to prevent the increase of an apoptotic factor (caspase-3) resulting from oxidative-nitrosative stress, as demonstrated in an olfactory bulbectomy induced rat model of depression (Rinwa et al., 2013). However, no evidence exists indicating any anti-apoptotic effects of curcumin as related to its anti-depression effects. In this study, we show that chronic curcumin treatment significantly suppressed neuronal apoptosis induced by inflammation in a CUMS-induced rat model of depression. These findings not only confirm the neuroprotective effect of curcumin in this CUMS-induced depression model but offer new insights into the mechanistic basis for this neuroprotection.

Recent evidence has indicated that depression is associated with structural and functional injury within specific brain regions (Goldwater et al., 2009; Gilabert-Juan et al., 2011; Oh et al., 2012). In the present study, we focused on the vmPFC, a critical brain region believed to be involved with depression. Neuroimaging studies revealed that the activity of vmPFC was reduced in depression (Takahashi et al., 2004). Moreover, alterations in the structure and function were also found within the vmPFC in response to chronic stress in animal models (Cook and Wellman, 2004; Radley et al., 2006; Holmes and Wellman, 2009; McLaughlin et al., 2009). In the present study, our results indicate that CUMS exposure induced neuronal apoptosis in the vmPFC as indicated by significant increases in the number of TUNEL positive apoptotic cells in this area. Because TUNEL assay may also label cells with DNA damage by other means than in the course of apoptosis, for example the necrotic cells, then we further detected the DNA breakage using TEM. Consistently, the apoptotic cells within the vmPFC of stressed rat showed ultrastructural changes involving nuclear damage, presenting as chromatic agglutination, margination and nuclear karyopyknosis. Such morphological results suggest that cell apoptosis, localized to specific brain regions, may be involved in the pathophysiology of depression. Of particular significance, our current results demonstrating a reversal in depression-like behavioral responses by curcumin were found to be accompanied with a significant amelioration of cell apoptosis within the vmPFC of CUMS-exposed rats. We also found that CUMS-exposure induced a significant overexpression of IL-1β which paralleled the neuronal apoptosis observed within the vmPFC, an effect which was alleviated by curcumin. Moreover, our results showed that the levels of interleukin-1β protein in serum of CUMS exposed rats were significantly increased, which suggest that the changes in peripheral cytokines are paralleled with the inflammatory state of the central nervous system. More interestingly, chronic administration of curcumin also attenuated the increased levels of IL-1β in peripheral blood. This is generally consistent with multiple previous studies which found an increased level of IL-1β in peripheral blood of depressed patients (Panighini et al., 2013; Su et al., 2017; Zou et al., 2018). It is important to note that the serum IL-1β may possibly led to the damage of blood-brain barrier and enhanced the inflammatory response centrally (Morgenstern and Pettigrew, 1997; Iadecola and Anrather, 2011; Labus et al., 2014). This overexpression of IL-1β may indicate one potential pathway through which neuroinflammatory mechanisms may proceed to produce neuronal apoptosis, eventually leading to depression. Accordingly, the neuroprotective ability of curcumin to regulate neuroinflammatory-induced neuronal apoptosis within the vmPFC indicates some of the underlying mechanisms of curcumin’s beneficial effects along with its potential for use as a novel tool in the treatment of depression.

The IL-1-type cytokines are major mediators of neuroinflammation and accumulating evidence suggests that IL-1β is a key contributor to neuronal deterioration in depression (Maes et al., 2012; Fernanda et al., 2017) Levels of IL-1β are increased within the brain in response to chronic stress (Pan et al., 2014) and inhibiting IL-1β reversed stress-induced social avoidance in rats (Ramirez et al., 2015). Accordingly, increased IL-1β expressions may play a critical role in inflammation-induced neuronal apoptosis after chronic stress. In the present study, we found that down-regulation of this IL-1β expression within the vmPFC of stressed rats significantly reversed cell apoptosis along with the display of depression-like behaviors, whereas overexpression of IL-1β within the vmPFC of unstressed rats significantly increased depression-like phenotypes. In order to determine whether curcumin protects against IL-1β-induced neuronal apoptosis, we infused IL-1β-expressing viruses into the vmPFC of unstressed rats to overexpress IL-1β and examined the effects on neuronal apoptosis and depression-like behaviors. The results of this experiment demonstrated that curcumin was effective in reducing the apoptotic and depression-like phenotypes induced by IL-1β overexpression within the vmPFC of rats. Consistent with these results were the findings showing that the downregulation of the pro-apoptotic proteins Bak, Caspase-3 and Caspase-9 and an upregulation of the anti-apoptotic protein Bcl-2 within the vmPFC of stressed rats. Therefore, these findings suggest that an anti-inflammatory strategy may contribute to antidepressant-like effects of curcumin via prevention of inflammation-induced neuronal apoptosis in response to chronic stress. The ensuing issue to address in these investigations involves identifying the downstream molecular targets of IL-1β in mediating vmPFC neuronal apoptosis in depression.

The MAPK pathway represents an important signal transduction pathway in the regulation of inflammation and apoptosis (Kim and Choi, 2015). It has been reported that p38 MAPK plays a major regulatory role in the crosstalk between the caspase-dependent pathway and apoptosis (Park et al., 2003; Wang et al., 2018). Our present results show that chronic stress significantly increased the expression of IL-1β and phosphorylation levels of p38 MAPK within the mPFC. With an intracerebral injection of AAV-IL-1β-RNAi virus into the vmPFC of stressed rats, as a means to block IL-1β expression and function, a significant reduction in p38 MAPK phosphorylation levels were observed. This effect paralleled the decreases in pro-apoptotic proteins levels suggesting that CUMS-induced neuroinflammation played a critical role in p38 MAPK phosphorylation. More importantly, the decreases in neuronal apoptosis within the vmPFC and depression-like behaviors induced by CUMS were also ameliorated by the injection of IL-1β-RNAi virus. Conversely, an overexpression of IL-1β as accomplished with an infusion of AAV-IL-1β virus into the vmPFC of unstressed rats up-regulated the activity of the p38 MAPK pathway and produced apoptotic phenotypes similar to that induced by chronic stress. However, a 2-week treatment of curcumin following AAV-IL-1β virus infusion significantly prevented the IL-1β/p38 MAPK pathway-induced neuronal apoptosis in the vmPFC along with a downregulation in the pro-apoptotic proteins Bak, Caspase-3 and Caspase-9 and an upregulation of the anti-apoptotic protein Bcl-2. Related results from a previous study have supported these findings as IL-1β has been shown to activate p38 MAPK cascades (Yu et al., 2017) and been shown to induce robust neuronal apoptosis resulting in neuronal injury within the rat hippocampus (Dong et al., 2017). Collating these findings, it seems likely that IL-1β-activated p38 MAPK cascades may participate in the ameliorative effects of curcumin on neuronal apoptosis and depression-like behaviors in our CUMS rats. In this way, IL-1β/p38 MAPK dysregulation may serve as an important trigger involved in the neurobiological and behavioral changes in stressed rats and that the antidepressant mechanisms of curcumin may result from its modulatory neuroprotective effects upon these pathways within localized depression-related sites of the brain. However, detailed molecular mechanisms regarding the means through which chronic stress may regulate IL-1β expression will require further investigation.

This study has some limitations. Firstly, it should be noted out that the nuuroinflammation theory of depression is only one hypothesis among several possibilities underlying the pathophysiological mechanisms. Moreover, only IL-1β was subjected to further investigation and analysis in this depression animal model, and the analysis of other pro-inflammatory factors would also be valuable. Additionally, the present study lacks of direct evidence to confirm the neuroinflammatory response act as a causative factor led to the depressive behaviors in rats. Therefore, whether and how curcumin exerts antidepressant-like effects via suppressing neuroinflammation remains further investigation. Finally, depression is a complex disease involving potential interactions among gene-environment and gene-gender factors. This study failed to consider gender factors, which could also influence the study results.

In summary, the present study revealed a potential novel neuroprotective mechanism whereby curcumin exerts antidepressant-like effects might via preventing inflammation-induced neuronal apoptosis in an animal model of depression. Our current results suggest that curcumin attenuates CUMS-induced neuronal apoptosis by inhibiting the IL-1β/p38 MAPK signaling pathway and regulating apoptosis-related protein expressions. Such findings not only reveal some of the underlying mechanisms for curcumin’s beneficial effects but also their potential for use as a therapeutic strategy against inflammation-related neuronal disorders.

## Author Contributions

CF, QS, PW, and SY contributed to the study design and analyses of data. CF performed the biochemical analysis and intracerebroventricular injections, immunohistochemistry and confocal imaging and Golgi staining experiments with QS. PW performed the synaptic image analysis. YL and MY performed depression model and behavioral tests. SY wrote the first draft. CF and QS participated in the subsequent drafts.

## Conflict of Interest Statement

The authors declare that the research was conducted in the absence of any commercial or financial relationships that could be construed as a potential conflict of interest.
